# High Physiological ^18^ F-FDG Uptake in Normal Pituitary Gland on Digital PET Scanner

**DOI:** 10.1055/s-0044-1786733

**Published:** 2024-05-01

**Authors:** Anjali Jain, Sharjeel Usmani, Khulood Al Riyami, Avni Mittal, Sofiullah Abubakar, Asiya Al Busaidi, Subhash Chand Kheruka, Rashid Al Sukaiti

**Affiliations:** 1 Department of Radiology & Nuclear Medicine, Sultan Qaboos Comprehensive Cancer Care, and Research Center, Muscat, Oman

**Keywords:** ^18^ F-FDG PET/CT, pituitary gland, digital PET, conventional PET

## Abstract

**Purpose** Recently developed digital positron emission tomography/computed tomography (PET/CT) scanners (digital PET [dPET]) have given new dimensions to molecular imaging. dPET scanner has very high sensitivity, spatial resolution, and image contrast that leads to increased uptake of signal in small-volume structures like pituitary gland (PG) making them visible on PET/CT scan even in absence of any pathology. Adequate knowledge of physiological fluoro-2 deoxy D glucose uptake in PG is required in interpretation of dPET for correct diagnosis and reducing unnecessary additional imaging. The aim of this study is to evaluate the frequency of physiological PG uptake on dPET.

**Material and Methods** Eighty-eight subjects (mean age, 54.44 ± 14.18 years; range, 26–84 years; 63 females and 25 males) with normal PG on magnetic resonance imaging brain and imaged within 6 months on dPET were included in this research study. Out of 88 patients, 20 control subjects (mean age, 58.15 ± 11.08 years: 15 females and 5 males) underwent PET/CT on conventional PET. All images were acquired with similar and standard acquisition protocol and reconstruction done with Time of flight with Point spread function. PG uptake was compared visually and quantitatively.

**Results** PG uptake was seen in 43 patients (48.8%). Out of 43 patients, 31 (72%) showed low uptake, 11 (26%) showed intermediate grade of uptake, and 1 patient (2%) showed intermediate-to-high uptake and was categorized as high-grade uptake. In the control group of 20 patients, 3 (15%) showed low uptake, while none of them showed intermediate or high uptake.

**Conclusion** Physiological PG uptake is commonly seen on dPET. Low-to-intermediate grade of PG uptake on dPET in an asymptomatic patient is physiological and does not require further evaluation and should be reported with caution.

## Introduction

Positron emission tomography/computed tomography (PET/CT) with ^18^ F-2 fluoro-2 deoxy D glucose ( ^18^ F-FDG) is routinely used for evaluation of various malignancies as is standard in clinical practice. It has incremental value over other imaging modalities like CT and magnetic resonance imaging (MRI) in diagnosing more sites of disease and upstaging the malignancy. Most PET/CT scanners available in various parts of the world are conventional (analog) scanners. The pituitary gland (PG) is situated in sella turcica in base of the skull. Due to the small volume of the normal PG, its uptake is similar to background activity on ^18^ F-FDG PET scan. 1 This underestimated uptake of FDG in small structures is due to partial volume effect, which makes the normal PG nonvisualized on routinely performed PET/CT on analog scanner. 2 3 4 Incidental physiological detection of PG on conventional ^18^ F-FDG PET/CT scan is a very rare finding and has been reported in less than 1% of cases in different studies. 5 Since physiological PG uptake is extremely rare, thus any uptake in PG warrants further clinical and radiological evaluation. 6 Majority of the cases with ^18^ F-FDG uptake in PG are due to pituitary pathology; common ones include pituitary adenoma, metastases, Langerhans cell histiocytosis, and hypophysitis. 7 8 Recently developed digital PET (dPET) scanners have given new dimensions to PET scans. dPET scanners include silicon photomultipliers (SiPMs) instead of regular photomultiplier tubes used in analog PET systems. 9 These smaller SiPMs provide 100% crystal area coverage leading to higher system sensitivity, spatial resolution, fast timing resolution, and low noise. Good image contrast and higher diagnostic performance also lead to reduced tracer dose and scan acquisition times. 10 11 Studies have shown that newly developed dPET scanner has approximately 70% increased sensitivity as compared to analog PET with a spatial resolution of approximately 3.7 mm. 10 This probably leads to apparent increase in physiological uptake in small-volume structures like the PG. There is increased standard uptake value (SUV) signal recovery, which makes the PG appear avid and unusual on dPET. This abnormal/avid looking normal PG may lead to unnecessary further imaging and clinical dilemmas. 12 Adequate knowledge of physiological PG uptake on dPET imaging is important as it will lead to correct interpretation of PET findings and reduce unnecessary additional imaging, which in turn will reduce radiation exposure, and cost and time for the patients and health care system. The aim of our study is to assess the frequency of high ^18^ F-FDG uptake of normal PG on dPET scanner and to evaluate the degree of physiological uptake and approximate SUV values of normal PG. The secondary objective is to compare the findings with the scan performed on analog PET scanner.

## Materials and Methods

### Patient Population

This retrospective, observational, cross-sectional study was conducted at Sultan Qaboos Comprehensive Cancer Care, and Research Center, Muscat, Oman (SQCCCRC). The study was approved by the hospital Medical Research Ethical Committee (IRB & EC Project ID CCCRC-45-2022) and informed consent was not required. All PET/CT scans done on dPET scanner between August 2021 and April 2022 were collected from the institution's Radiology information system (Philips Health Care System). After computerized search of the institutional radiology database, a total of 477 ^18^ F-FDG PET patients were recorded after excluding ^68^ Ga (DOTA0-Phe1-Tyr3) octreotide (DOTA)/Prostate-specific membrane antigen (PSMA) PET studies and repeat studies. Final study population was segregated by Nuclear Medicine physician based on inclusion and exclusion criteria.

*Inclusion criteria* : (1) ^18^ F-FDG PET/CT scans done on dPET scanner with an MRI of the brain done at SQCCCRC within 6 months of PET/CT scan. (2) All ages and both gender patients were included.

**Fig. 1:**
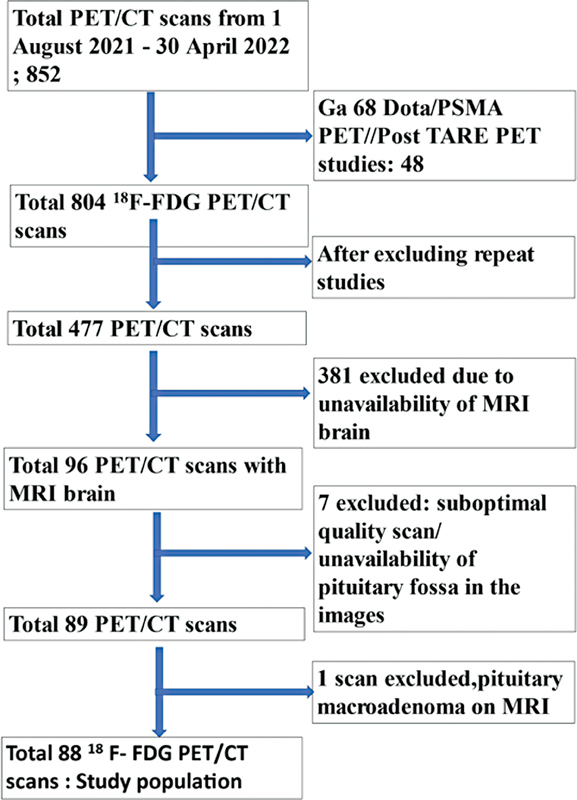
Patient enrolment chart. ^18^ F-FDG PET/CT, ^18^ F-2 fluoro-2 deoxy D glucose positron emission tomography/computed tomography; MRI, magnetic resonance imaging.

*Exclusion criteria* : (1) Unavailability of MRI brain within 6 months of PET/CT. (2) Suboptimal image quality (significant muscle uptake due to insulin and scans, which do not include pituitary fossa in the images). (3) Patients with PG pathology on MRI. Finally, 88 patients (PET/CT scans done on digital scanner) were included as study population ( Fig. 1 ).

### ^18^ F-FDG PET/CT

All ^18^ F-FDG PET/CT scans were acquired with standard departmental protocol on dPET with 128 slice CT scanner (Siemens Biograph Vision 600, Siemens Healthineers). Patient preparation was as per standard guidelines, including fasting for 6 hours with restriction of intravenous (IV) dextrose drip and insulin 6 hours before the study. Patients were instructed to avoid exercise for 24 hours. Fasting blood sugar was less than 11.1 mmol/L for all scans. ^18^ F-FDG dose administered was 2 to 3 MBq/kg. IV contrast was not given, and water was used as negative oral contrast. Total uptake time for all studies was 60 minutes ± 10 minutes after tracer administration. Whole-body low-dose, nonbreath-hold CT was acquired first, followed by PET images with three-dimensional emission scan with slice thickness of 5 mm. Low-dose CT was used for attenuation correction and anatomical localization. PET and CT images were reconstructed using OSEM + Time of flight (TOF) + Point spread function (PSF) (True X). Iteration, 4; Subsets, 5; Filter Gaussian, Full width at half maximum (FWHM) (mm) 2; Matrix, 440; Zoom, 1; and fused images were generated in the system. All images were transferred to Philips picture archiving and communication system (PACS) system for interpretation by a Nuclear Medicine physician.

In addition, 20 patients out of total 88 patients also underwent ^18^ F-FDG PET/CT on conventional PET (cPET) scanner during their follow-up. These 20 patients with ^18^ F-FDG PET/CT scans done on a conventional scanner (Biograph mCT flow with 128 slice CT scanner, Siemens Healthcare, Erlangen, Germany) were included in the study as a control group for comparison. The scan was acquired on analog PET scanner with similar, standard patient preparation and protocol and reconstruction parameters. The administered ^18^ F-FDG doses were 3.7 to 5.2 MBq/kg.

## Image Interpretation and Quantitative Analysis

**Fig. 2:**
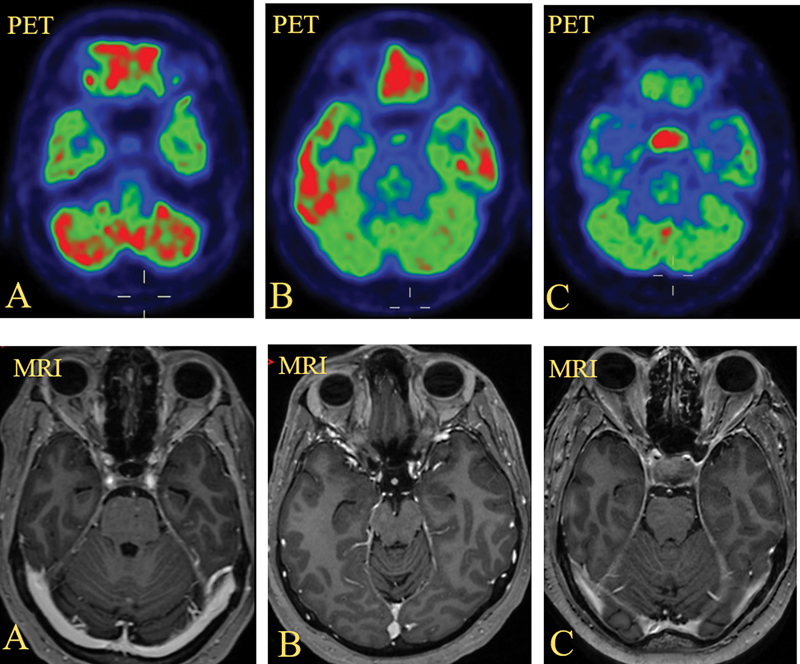
Grades of pituitary uptake. ( **A** ) Positron emission tomography (PET) and magnetic resonance imaging (MRI) images of a 40-year-old female, with the case of breast cancer on hormonal treatment; 18F-2 fluoro-2 deoxy D glucose ( ^18^ F-FDG) PET/computed tomography (CT) scan was done for restaging. Low uptake is seen in normal pituitary gland. ( **B** ) PET and MRI images of a 35-year-old female, with the case of choriocarcinoma with lung metastases; ^18^ F-FDG PET/CT scan was done for staging. Intermediate uptake is seen in normal pituitary gland. ( **C** ) PET and MRI images of a 64-year-old male, with the case of colon cancer; ^18^ F-FDG PET/CT scan was done for staging. High uptake is seen in pituitary gland, and corresponding MRI images show pituitary macroadenoma.

All PET/CT scans were analyzed qualitatively and quantitatively. For visual assessment, the scans were first optimized for intensity and set at a scale of 0 to 10 on the PET component of the study. The images at the level of pituitary fossa/PG were reviewed. Any focal uptake in the PG was considered as positive and absence of focal uptake with uptake equal to background was considered as negative for PG uptake. Using a 3-point color bar in the Philips PACS system, the uptake in pituitary was graded as low, intermediate, or high. The gray matter of the cortex was seen to vary between green and red colors on the 3-point color bar. Uptake in PG less than outer gray matter of brain was considered as low uptake (pituitary uptake seen in blue color) ( Fig. 2A ). Uptake equal to gray matter was graded as intermediate uptake (pituitary uptake seen in green color) ( Fig. 2B ) and any uptake more than gray matter of brain (pituitary uptake seen as red color) was recorded as high uptake ( Fig. 2C ). The percentage of patients with positive PG uptake was calculated. In the positive group, the percentage of patients with low, intermediate, and high-grade uptake was calculated.

Quantitative assessment of PG was made by measuring the SUVs. SUV max and SUV mean for the PG were measured by drawing a regions of interest (ROI) (isocontour ROI) on the pituitary fossa. Similarly, SUV max and SUV mean were also calculated for the background in the skull region (seen in black color outside gray matter of brain on 3-point color bar) ( Fig. 2 ) and in the mediastinal blood pool. The mean and standard deviation (SD) of SUV max and mean were recorded and ratios of pituitary uptake with background and with mediastinum were calculated.

The visual and quantitative analyses of the 20 control patients, performed on an analog scanner, were performed in a similar manner. The two sets of images with dPET and cPET were compared.

### Statistical Analysis

Statistical analysis was performed using the statistical package for the social sciences (SPSS 30.0, Chicago, Illinois, United States). Results were presented as mean ± SD. Nonparametric test (Mann–Whitney *U* -test) was applied to compare the data between dPET and cPET. *p* -Value ≤ 0.05 is considered significant.

## Results

A total of 88 patients fulfilled the inclusion criteria and were included as our study population. The mean age of the subjects (imaged on dPET) included in the study was 54.44 ± 14.18 years (range: 20–82 years; 25 males and 63 females). The control group (imaged on conventional scanner) consisted of 5 men and 15 women with mean age 58.15 ± 11.08 years. Patient characteristics are presented in Table 1 .

**Table 1:** Patient characteristics

	Mean ± SD	
	Control ( *n* = 20)	Subjects ( *n* = 88)
Age	58.15 ± 11.08 years	54.44 ± 14.18 years
Female:Male	15:5	63:25
Type of cancer		
Breast	10	43
Lung	3	12
GI malignancy	2	17
Hepatobiliary	2	5
Others	3	11

Abbreviations: GI, gastrointestinal; SD, standard deviation.

**Fig. 3:**
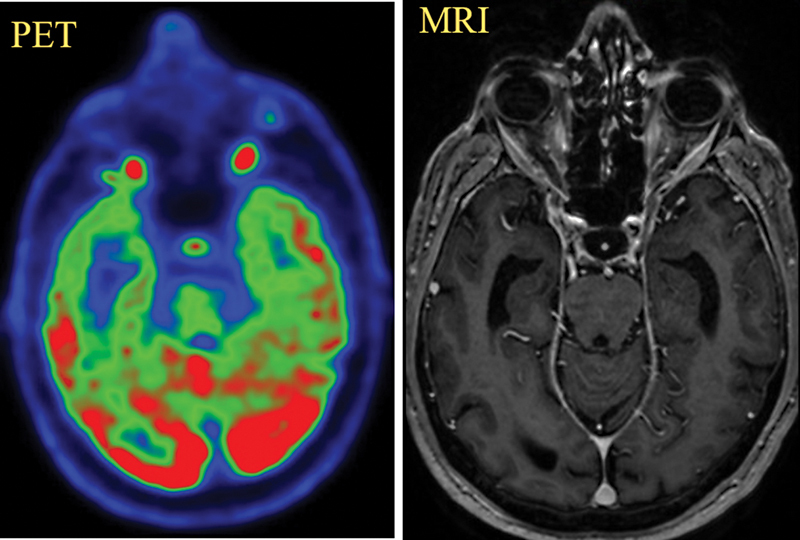
Positron emission tomography (PET) and magnetic resonance imagings (MRIs) of a 71-year-old male, with the case of acral melanoma; 18F-2 fluoro-2 deoxy D glucose PET/computed tomography scan was done for staging. Intermediate to high uptake is seen in normal pituitary gland.

Visual analysis of PG in our study population of PET/CT scan done on dPET system showed focal increased ^18^ F-FDG uptake in 43 out of 88 patients (49%). Forty-five patients (51%) did not show any increased uptake or visualization of the PG. Out of 43 patients who showed increased uptake in PGs, 31 patients (72%) showed low uptake (31 out of total study population of 88), 11 patients (26%) showed intermediate grade of uptake (11 out of total study population of 88), and 1 patient (2%) showed intermediate-to-high uptake visually and was categorized as high uptake (pituitary uptake seen in mixed red and green color) ( Fig. 3 ). All the patients included in our study showed normal PG on MRI. There was no difference in PG findings on MRI between low and intermediate uptake on dPET. None of these patients showed PG pathology on brain MRI. One patient showing intermediate-to-high uptake also did not show any pituitary pathology in the initial scan and at follow-up ( Fig. 3 ). One patient was found to have pituitary macroadenoma on brain MRI and was excluded from the study; however, it showed significantly increased FDG uptake with a very high SUV max of 17.0 ( Fig. 2C ).

In the control group of 20 patients, with PET/CT performed on an analog scanner, PG uptake was seen in 3 patients (15%). The rest of the 17 patients (85%) did not show any increased PG uptake. Out of three patients, all of them (100%) showed low-grade PG uptake and none of them showed intermediate or high uptake. PG was normal in all of them ( Table 2 ).

**Table 2:** Visual/Qualitative analysis of pituitary uptake on dPET and cPET

	Mean ± SD	
	Control ( *n* = 20)	Subjects ( *n* = 88)
Pituitary uptake (visual analysis)		
Yes	3 (15%)	43 (49%)
No	17 (85%)	45 (51%)
Grade of uptake		
No uptake	17	45
Low uptake	3 (100)	31 (72%)
Intermediate uptake	0	11 (26%)
High uptake	0	1 (2%)

Abbreviations: cPET, conventional positron emission tomography; dPET, digital positron emission tomography; SD, standard deviation.

On quantitative analysis, the mean of SUV max in patients on dPET with focal increased PG uptake was 4.63 ± 1.11 as compared to mean of SUV max 2.67 ± 0.57 ( *p* < 0.001) in patients with no PG uptake. The other parameters, pituitary SUV mean and pituitary/background ratios in SUV max and SUV mean, are mentioned in Table 3 .

**Table 3:** Quantitative analysis of pituitary/grades uptake seen on digital PET

	*n*	Pituitary SUV max	*p* -Value	Pituitary SUV mean	*p* -Value	Pituitary/background (SUV max)	*p* -Value	Pituitary/background (SUV mean)	*p* -Value
Visual analysis									
No uptake	44	2.67 ± 0.57	<0.01	1.92 ± 0.52	<0.01	2.86 ± 1.19	<0.01	3.18 ± 1.34	<0.01
Uptake	43	4.63 ± 1.11		3.04 ± 0.75		4.55 ± 1.45		4.68 ± 1.40	
Grade of uptake									
No uptake	45	2.67 ± 0.57		1.92 ± 0.52		2.86 ± 1.19		3.18 ± 1.34	
Low uptake	31	4.06 ± 0.65		2.74 ± 0.55		4.36 ± 1.55		4.50 ± 1.51	
Intermediate uptake	11	5.95 ± 0.44		3.77 ± 0.66		4.99 ± 1.14		5.13 ± 0.98	
High uptake	1	7.60		4.40		5.428			

Abbreviations: PET, positron emission tomography; SUV max, maximum standardized uptake value.

The pituitary SUV max, SUV mean, and pituitary/background ratios in different grades of uptake are mentioned in Table 3 .

Patients imaged with dPET presented with higher pituitary SUV max and SUV mean compared to patients imaged with cPET (3.63 ± 1.31 vs 2.63 ± 0.51, *p* = 0.0011; and 2.47 ± 0.85 vs 1.99 ± 0.46, *p* = 0.012, respectively) ( Table 4 ). The pituitary/background (SUV max) is higher in dPET (3.68 ± 1.57) compared to cPET (2.85 ± 0.74) ( *p* = 0.030), while there was no significant difference for pituitary/background SUV mean (3.91 ± 1.56 vs 3.27 ± 0.97; *p* = 0.098).

**Table 4:** Quantitative analysis of pituitary uptake in digital PET and conventional PET

Quantitative parameter	Mean SUV ± SD		*p* -Value
	Control ( *n* = 20)	Digital PET ( *n* = 88)	
Pituitary (SUV max)	2.63 ± 0.51	3.63 ± 1.31	0.001
Pituitary (SUV mean)	1.99 ± 0.46	2.47 ± 0.85	0.012
Background (SUV max)	0.955 ± 0.19	1.07 ± 0.34	0.116
Background (SUV mean)	0.63 ± 0.13	0.67 ± 0.20	0.376
Mediastinal (SUV max)	2.32 ± 0.51	2.65 ± 0.56	0.021
Mediastinal (SUV mean)	1.78 ± 0.38	1.95 ± 0.42	0.114
**Target to background ratio**			
Pituitary/background (SUV max)	2.85 ± 0.74	3.68 ± 1.57	0.030
Pituitary/background (SUV mean)	3.27 ± 0.97	3.91 ± 1.56	0.098

Abbreviations: PET, positron emission tomography; SD, standard deviation; SUV max, maximum standardized uptake value.

Mann–Whitney *U* -test: the significance level is 0.05.

The pituitary/mediastinal SUV max and SUV mean were not significant with *p-* value more than 0.05.

## Discussion

Incidental physiological visualization of PG uptake on conventional FDG PET/CT scan is a very rare finding and has been reported in less than 1% of cases in different studies. 13 Hyun et al 14 in their study on 13,145 subjects found that incidental pituitary uptake was seen in 0.8% of subjects in which 41% were pathological. They suggested that incidental, focal, pituitary uptake with SUV max more than 4.1 should be further investigated with pituitary MRI and/or testing of hormonal levels or endocrinology review.

^18^ F-FDG scans performed on dPET scanners have higher sensitivity, resolution, and image contrast due to their technology. This leads to increased visualization of small-volume structures like PG. Higher count rate detection efficiency on a dPET with true digital photon counting with 1-to-1 crystal coupling leads to visualization of small structures, further leading to false positive results. 15 16

Our study of 88 patients with ^18^ FDG PET/CT performed on digital scanner showed increased uptake/visualization of normal PG in 43 (49%) patients, which is significantly more than our control group of 20 patients done on conventional scanner, where physiological pituitary uptake was seen in only 15% of subjects.

To the best of our knowledge, there is only one study published 12 so far comparing PG uptake in dPET vs cPET and only one conference abstract 17 that has compared physiological pituitary uptake in dPET with cPET. The results of these studies are like our study. The conference abstract by Manabe et al with a retrospective study on 45 patients has shown that on dPET focal PG FDG accumulation was seen in 23 of 45 patients (52.3%), which was significantly higher compared to that on cPET in 5 of 41 patients (12.2%) ( *p* = 0.0001). 17 The results of their study are very close to our findings. Similarly, Meyer et al 12 conducted a study on 10 dPET and 10 cPET scans and found that PG hypermetabolism was seen in 80% of patients with dPET as compared to 10% in cPET. They showed approximately similar number of patients with pituitary hypermetabolism on cPET, although the percentage of patients with pituitary hypermetabolism on dPET was higher as compared to our study. This could possibility be due to their smaller sample size, different reconstruction parameters, etc.

In our study, we have also shown that the mean of SUV max in patients with focal increased PG uptake on dPET is higher (4.63 ± 1.11) as compared to mean of SUV max on cPET (2.67 ± 0.57) with significant value ( *p* < 0.001). This further confirms our visual interpretations.

In an effort to find out the degree of uptake that can be attributed to be normal or physiological, we have in addition further divided our patient population with increased PG uptake into three categories of low, intermediate, and high. In our study we have seen that majority of patients (72%) show low-grade uptake and a few of them (26%) show intermediate-grade uptake. One patient (2%) also showed intermediate-to-high-grade uptake. One patient with macroadenoma in PG showed very high uptake and was excluded from the study. No other patient with normal PG showed high FDG uptake. In the control group, all 15% of patients showed low uptake and none of them demonstrated intermediate or high uptake. To the best of our knowledge, none of the prior studies in literature have shown this.

While interpreting PG uptake, it is important to know which grade of uptake can be believed to be physiological, and this information is crucial to segregate normal and abnormal PG uptake while reporting. As seen in our study, physiological PG uptake in dPET is a common finding and is low in most of the scans. Intermediate uptake can be seen in few of the patients with normal PG with dPET. These patients do not require any further imaging or evaluation. Except for one patient with borderline high uptake, no other patient showed high or significant uptake. Therefore, any high PG uptake, more than outer gray matter of brain, should be further evaluated to exclude pituitary pathology.

SUV values depend on many factors and change with reconstruction parameters, as also shown in various studies. 18 19 In our study with dPET and TOF with PSF reconstruction, we have seen that SUV max and SUV mean of PG imaged with dPET were higher and significant when compared to patients imaged with cPET (3.63 ± 1.31 vs 2.63 ± 0.51, *p* = 0.0011; and 2.47 ± 0.85 vs 1.99 ± 0.46, *p* = 0.012, respectively). Also, the pituitary/background (SUV max) was higher in dPET (3.68 ± 1.57) compared to cPET (2.85 ± 0.74) ( *p* = 0.030), although there was no difference for pituitary/background (SUV mean) for digital vs conventional scans, with 3.91 ± 1.56 vs 3.27 ± 0.97, respectively ( *p* = 0.098). These results are also similar to the previous study and abstract in the literature where Meyer et al 12 have shown that patients imaged with dPET presented higher PG SUV max and SUV ratio compared to patients imaged with cPET (4.7 ± 2.05 vs 2.9 ± 0.64, *p* = 0.004; and 0.62 ± 0.25 vs 0.39 ± 0.09, *p* = 0.029, respectively), while there was no difference for SUV mean (2.7 ± 1.32 vs 2.1 ± 0.44, *p* = 0.39). 12 Similarly, Manabe et al also showed that pituitary SUV max was significantly higher on dPET as compared to cPET (4.1 ± 3.9 vs 2.5 ± 0.6, *p* < 0.0001). 17 There was no significant difference seen in background and mediastinal SUV max and mean between dPET and cPET scans in our study.

The limitations of our study are the relatively small number of studied patients and a single-center study. SUV values are based on reconstruction parameters followed at our institute as per our protocol and it may vary in different institutions depending on reconstruction parameters and other factors. Additionally, the control group was smaller than the study population. Out of 88 patients, only 20 patients had a previous ^18^ F-FDG PET/CT scan done on conventional scanner. Since it was good match in terms of age and other factors and as up to 4:1 ratio of case vs control is acceptable, 20 a comparison of case vs control was included as part of the study.

## Conclusion

PG uptake is commonly seen on dPET. Low-to-intermediate grade of PG uptake on dPET could be physiological with no requirement for further evaluation. These should be reported with caution. A high-grade ^18^ F-FDG uptake in PG should be evaluated further with an MRI brain and biochemical evaluation to exclude pituitary pathology. Further work is needed to evaluate high physiological uptake in other small-volume structures on dPET scanner.
